# Biofilm Research in Bovine Mastitis

**DOI:** 10.3389/fvets.2021.656810

**Published:** 2021-05-07

**Authors:** Regitze Renee Pedersen, Volker Krömker, Thomas Bjarnsholt, Kirstin Dahl-Pedersen, Rikke Buhl, Elin Jørgensen

**Affiliations:** ^1^Department of Veterinary Clinical Sciences, University of Copenhagen, Copenhagen, Denmark; ^2^Department of Veterinary and Animal Sciences, University of Copenhagen, Copenhagen, Denmark; ^3^Department Immunology and Microbiology, Costerton Biofilm Center, University of Copenhagen, Copenhagen, Denmark; ^4^Department of Clinical Microbiology, Copenhagen University Hospital, Copenhagen, Denmark

**Keywords:** biofilm, bovine mastitis, chronic infections, antimicrobial resistance, antimicrobial therapy

## Abstract

Bovine mastitis is one of the most important diseases in the dairy industry and has detrimental impact on the economy and welfare of the animals. Further, treatment failure results in increased antibiotic use in the dairy industry, as some of these mastitis cases for unknown reasons are not resolved despite standard antibiotic treatment. Chronic biofilm infections are notoriously known to be difficult to eradicate with antibiotics and biofilm formation could be a possible explanation for mastitis cases that are not resolved by standard treatment. This paper reviews the current literature on biofilm in bovine mastitis research to evaluate the status and methods used in the literature. Focus of the current research has been on isolates from milk samples and investigation of their biofilm forming properties *in vitro*. However, *in vitro* observations of biofilm formation are not easily comparable with the *in vivo* situation inside the udder. Only two papers investigate the location and distribution of bacterial biofilms inside udders of dairy cows with mastitis. Based on the current knowledge, the role of biofilm in bovine mastitis is still unclear and more *in vivo* investigations are needed to uncover the actual role of biofilm formation in the pathogenesis of bovine mastitis.

## Introduction

Bovine mastitis is an important disease in the dairy industry with severe consequences for the welfare of dairy cows and the economy of the industry (1). Antibiotic treatment of bovine mastitis account for the highest antibiotic use in the dairy industry (2).

Bovine mastitis is defined as inflammation of the mammary gland and is most commonly caused by bacterial infection (3). Bovine mastitis occurs in two different clinical manifestations; subclinical and clinical mastitis, and ranges from mild, moderate to severe cases. Subclinical mastitis can be diagnosed by tests, e.g., the somatic cell count in milk, however, no clinical signs are apparent (4). Clinical mastitis manifests with visible changes to the milk in the form of clots or flakes and clinical signs of infection and inflammation, such as fever, redness, pain, and swelling of udder and lymph nodes (4). Some cases of bovine mastitis resolve themselves and most cases resolve after standard antibiotic treatment (2), however, some cases can progress to a detrimental point where the cow is culled, and in severe cases, spontaneous death may even occur (1, 4).

The most common infectious agents of bovine mastitis are *Staphylococcus aureus, Streptococcus agalactiae* (2), *Escherichia coli* (5), and *Streptococcus uberis* (6). *S. aureus* is a common and challenging mastitis pathogen, as *S. aureus* has a high persistence rate (7, 8) and a low bacteriological cure rate in clinical mastitis cases (9). During bovine mastitis, bacteria potentially upregulate expression of virulence factors that can lead to higher resistance to phagocytosis (10) and upregulation of genes that destruct host tissue and the ability of the host cells to capture iron, e.g., lactoferrin (11). The pathogens are adapted to infection of the tissue in the mammary gland by a broad variety of virulence factors, e.g., the propensity to invade and escape host cell defenses by hemolysins (12), adhesion to host cells and production of leukotoxins to destroy monocytes and polymorphonuclear cells (12). Further, some of the pathogens are low shedders (13) and some form biofilm (12). All resulting in pathogens capable of causing long-lasting infections.

Bovine mastitis is normally treated with antibiotics, however, in some cases, the antibiotics are not resolving the disease and the infection becomes chronic. Continued antibiotic treatment in these cases where antibiotics do not eradicate the microbial agents increases the risk of developing antibiotic resistance, which is one of the greatest threats to human and animal health (14).

Chronic and recurrent cases of bovine mastitis share similar characteristics with chronic biofilm infections observed in humans and other animals. Biofilm is defined as “a coherent cluster of bacterial cells imbedded in a biopolymer matrix, which, compared with planktonic cells, shows increased tolerance to antimicrobials and resists the antimicrobial properties of the host defense” (15), however the role of matrix is unclear *in vivo* (16). Biofilm is suggested to be the default mode of growth for bacteria (17). Antibiotic treatment of biofilm infections is often unsuccessful and thus the infections are difficult to eradicate (18, 19). Being part of a biofilm can provide protection for the bacteria against threats from the environment, including antibiotics and host defenses (20).

The role of biofilm in human infections has been an expanding research field since bacterial aggregates were observed in 1977 in the lungs of patients with cystic fibrosis (21), and since 1982 where the first report on a medical biofilm causing recurrent infection (bacteremia) was described (22). However, in veterinary medicine, few reports exist on biofilms' direct role in infections, as most literature focuses on *in vitro* characteristics of pathogenic bacteria/biofilms and not their role *in vivo*.

In human medicine, biofilms are known to contribute to a wide variety of infections and diseases including wound infections, implant related infections, lung infections, osteomyelitis, chronic otitis media, urinary tract infections, chronic sinusitis, dental plaque, endocarditis, etc. (23). *Pseudomonas aeruginosa* biofilm infection in cystic fibrosis patients is one of the most well-studied biofilm infections to date, and intense research has revealed both pathogenetic, diagnostic, and therapeutic breakthroughs, and has increased the life expectancy of these patients dramatically (24–26). Biofilms are found in the majority of human chronic wounds and are considered to play a consistent role in the pathogenesis of impaired wound healing (27–29). Major biofilm pathogens in chronic wounds are *S. aureus, P. aeruginosa*, and *Enterobacteriaceae* (27, 30, 31). Implant related infections are often also driven by biofilms that cause low grade, difficult to detect infections with delayed onset (32, 33). Biofilm infections are thus important and are estimated to account for 550,000 deaths and 17 million infections yearly in the USA (34).

Understanding the role of biofilm in bovine mastitis will potentially unlock new treatment options and avoid unnecessary antibiotic treatment. If thereby being able to cure these chronic and recurrent bovine mastitis cases, the economy of the dairy industry, as well as animal welfare will improve and use of antibiotics will decrease.

In this paper, we review the literature on the development of bovine mastitis biofilm research with focus on the last two decades. In addition, we review the methods applied in published research and propose new methods for future research of biofilms' role in bovine mastitis.

## Development of Research of Bovine Mastitis

The first studies investigating biofilm forming abilities of bovine mastitis pathogens emerged in the early 1990s. In 1993, “slime production” (exopolysaccharide matrix) was observed in bovine coagulase-negative staphylococci (CNS) strains. This slime production was observed *in vitro* by using the tube method together with Congo Red Agar plates and suggested that the slime-production functioned as a virulence factor (35). Later, strains of *S. aureus* isolated from bovine mastitis cases were found to bind to milk fat globules. This suggested that the bacteria were in a biofilm mode of growth *in vitro* (36). During the first decade of 2000, most papers concentrated on investigating the *in vitro* biofilm forming abilities of *S. aureus* and *Staphylococcus epidermidis* isolates from bovine mastitis cases (37–39), the genes that were associated with biofilm formation (39–41), the susceptibility to antimicrobial agents (42), and potential treatment options against biofilm infections (43–45).

After 2010, the research of biofilm in bovine mastitis accelerated, and during the last decade, over 170 studies have been published. The focus of the research is still the *in vitro* biofilm forming abilities of bovine mastitis pathogens but also investigations of antibiotic resistance, molecular investigations of biofilm related genes, and the search for potential treatments and vaccines; the majority of these paper have focused on *S. aureus*. In only two *in vivo* studies, bacterial biofilms have been directly identified in bovine udders with mastitis (46, 47).

## Biofilm Methods Applied to the Research of Biofilm in Bovine Mastitis

### Methods Used for Investigation of Biofilm Forming Abilities of Bovine Mastitis Pathogens

The biofilm forming abilities of bovine mastitis pathogens *in vitro* have been investigated by multiple traditional biofilm methods. Most studies have focused on bacterial isolates from milk samples of bovine mastitis cases and the main focus has been on *S. aureus*, a well-known *in vitro* biofilm producer (48) and one of the most common pathogens in chronic bovine mastitis (49). The majority of the studies, i.e., more than 140 papers, have been using microtiter plates with crystal violet staining for quantification of the bacterial biomass. When using this biofilm assay, the bacteria are grown in polystyrene microtiter plates. The wells are emptied and washed at different time points, whereby the remaining biofilm biomass can be stained and quantified with crystal violet (50, 51). The crystal violet stain is used to quantify the total biomass in these system, as the stain binds to negatively charged molecules, which means to both the bacteria and exopolysaccharides (50). *S. aureus* is the most common species investigated using the microtiter assay in biofilm and bovine mastitis research to investigate its ability to form biofilm *in vitro*. Multiple studies found that majorities of *S. aureus* isolates from bovine mastitis cases can form biofilm *in vitro* by this assay (52–54). Applying the same method, 20–30% of *S. agalactiae* mastitis isolates also showed biofilm forming abilities *in vitro* when cultivated in different atmospheric conditions and growth media (55–57). This assay was also used to investigate the biofilm forming abilities of 53 mastitis isolates of *Klebsiella* spp. and 17 *Pseudomonas aeruginosa* mastitis isolates, all isolates were able to form biofilm (58, 59).

Although not as common as the crystal violet assay, several studies use the Congo Red Agar (CRA) test. The CRA method was developed by Freeman et al. (60) in 1989 for “detecting the production of slime by coagulase-negative staphylococci.” The “slime-forming” strains are black and the strains not capable of forming slime appear red on the agar (60). The CRA test is a qualitative method to estimate whether staphylococci isolates are able to produce biofilm *in vitro* and is often followed by a quantitative assay—such as the tube method or the microtiter assay. Half of *S. aureus* isolates from dairy cows with subclinical mastitis were able to produce biofilm by the CRA method (61, 62).

In the standard tube method, bacteria are cultivated in culture tubes, washed and then stained with crystal violet, safranine, or other stains. Biofilm production is observed by color on the sides and bottom of the tube (63). When the biofilm forming ability of *S. aureus* isolates from bovine mastitis cases was investigated by the tube method using safranine stain, 25–70% of the isolates were able to form biofilm (61, 64, 65).

Using yet another staining method, ~85% of CNS isolates from mastitis milk samples were able to form biofilm when their biofilm forming ability was investigated by the microtiter assay and stained using the LIVE/DEAD technique with subsequent confocal laser scanning microscopy (CLSM) to study the composition of the matrix (66). Confocal laser scanning microscopy is widely used in the visualization of medical biofilm, as some of the advantages of this technique are the possibility to visualize 3D and spatial structures of biofilms (51). Furthermore, it is possible to quantify volume and other parameters of the biofilm and to apply different fluorescent probes (51).

Quantitative and qualitative assays for investigating the biofilm forming abilities of bovine mastitis pathogens *in vitro* are inexpensive, fairly simple and fast. In the last years, microscope techniques have become more accessible and would facilitate more detailed investigations of the biofilm phenotype and interactions between antimicrobial compounds and biofilms.

### Investigations of Antimicrobial Compounds Against Biofilm Forming Mastitis Pathogens

Different antimicrobial compounds and antibiotics have been tested on bovine mastitis isolates' ability to form biofilm. The biofilm forming ability of *E. coli* in the presence of different antibiotics was investigated using CLSM and revealed increased adhesion of the isolates (67) and a greater biofilm formation of *E. coli* bovine mastitis isolates in the presence of enrofloxacin (68). When grown as biofilms, *S. aureus* bovine mastitis isolates are highly resistant to antimicrobial agents (42). The antibacterial use of the traditional medicinal plant *Plectranthus ornatus* (spur flowers) used in Brazil for treatment of skin infections was investigated for its anti-biofilm properties by using the plant as a herbal soap on gloves contaminated with *S. aureus* from dairy cows with bovine mastitis. There was no microbial growth after the gloves were submerged in the herbal soap and when the biofilm inhibitory concentration by microtiter plates and crystal violet staining was investigated, the plant was able to inhibit biofilm formation (69). Anti-biofilm agents against *S. aureus* have also been investigated *in vivo*. Ethanolic extracts from the leaves of *Rhodomyrtus tomentosa* (*rose myrtle*) were investigated as a possible antimicrobial agent against biofilm producing *S. aureus* in combination with the antibiotic pirlimycin. When extracts were used alone, there was no significant reduction in the bacterial load in a murine mastitis model. In combination with the antibiotic, a significant antibacterial effect was observed, but there was no significant difference between the antibiotic used alone compared with the combination of antibiotics and extract (70). The possible inhibitory effect of the Argentinian medicinal plant *Minthostachys verticillata* was tested on *Escherichia coli, Bacillus pumilus*, and *Enterococcus faecium* isolated from mastitis milk. The essential oil of the plant had inhibitory effect on the production of biofilm of all isolates in 96 well-microtiter plates (71). The naturally occurring signaling molecule of bacteria, cyclic dinucleotide 3′,5′-cyclic diguanylic acid (c-di-GMP), has been investigated to inhibit biofilm formation of *S. aureus*, and a decrease in the colonization of the pathogen in the mammary glands was shown in a murine mastitis model (44). The alternative drug, 1-hydroxyanthraquinone, was found to have a significant inhibitory action against *Staphylococcus xylosus in vitro* as well as a reduction in inflammation in the mammary glands of murine models (72).

### Biofilm-Associated Genes in Bovine Mastitis Pathogens

The molecular identification of pathogens is another direction in the research of biofilm in bovine mastitis and several studies have investigated different biofilm-associated genes of bovine mastitis isolates. The intercellular gene cluster adhesion operon (*ica*) is one of the genes that has been investigated for its role in biofilm formation and has been found in 40% of *S. aureus* isolates from bovine mastitis by analyzing their biofilm forming abilities within the microtiter assay and then sequencing the isolates (73). However, whether the isolates carrying the *ica* genes actually produce biofilm *in vitro*, depends on the biofilm assay. Some studies found that even if the isolates carried the *ica* genes, not all of the isolates produced biofilm in the microtiter plate (74) and that some isolates would form black colonies (indicating slime-formation) when grown on CRA plates but not necessarily form biofilm in the microtiter assay (41). Biofilm-associated proteins (bap) has been researched by several bovine mastitis studies and *S. aureus* isolates have been investigated for the presence of *bap* genes and their biofilm forming ability (40). A study found that over 90% of isolates carried *icaADBC* genes and of these 25% carried the *bap* genes. When the isolates were positive for both *icaADBC* and *bap*, they were strong biofilm producers *in vitro*, however, when only positive for *icaADBC*, they produced less biofilm. The role of *bap* was investigated by constructing a mutant only positive for *bap* and found that the mutant had the same biofilm forming capacity as the wild type (40). However, in other studies, the *bap* gene was not found at all in *S. aureus* isolates from bovine mastitis cases (39, 74).

## Other Topics Addressed in the Research of Biofilm in Bovine Mastitis

### Multispecies Biofilm

The research of bovine mastitis and biofilm often focuses on one specific pathogen and its ability to form biofilm *in vitro*. When only single species are investigated, there is a risk of overseeing keystone species (75) or possible interactions between commensals and pathogens or amongst pathogens, which might be important in the understanding of biofilms' role in bovine mastitis. However, the majority of studies investigating the role of biofilm in bovine mastitis focuses solely on *S. aureus*. In the environment, there is often more than one bacterial strain present and multispecies biofilms are commonly observed (76). Bovine mastitis infections can have multiple bacterial agents (9) and it is also important to consider the possible role of commensal bacteria in udders. Lactic acid bacteria (LAB) are commonly isolated from the teat canals and milk of dairy cows (77). Wallis et al. investigated the effect of growing two probiotic LAB strains together with a challenge between biofilm of probiotic LAB and *S. aureus* biofilm. They observed that when two LAB strains were co-cultured with *S. aureus*, it resulted in no growth of *S. aureus*, suggesting the beneficial use of probiotic bacteria against pathogenic biofilms in bovine mastitis (78). The presence of specific bacteria can either promote or decrease growth of other bacteria (79, 80) and the competition between these bacteria can cause damage to the surrounding environment or tissue (81). Immune responses toward bacteria and biofilm may similarly cause collateral damage to the surrounding tissue (82). Therefore, it is important to consider possible interactions between other bacteria and bovine mastitis pathogens as well as between pathogens and the immune response.

### Potential Vaccines Against Biofilm Forming Mastitis Pathogens

Two mastitis vaccine candidates against *S. uberis* have shown a significant reduction in the mortality of mice infected with the pathogen (83). Different candidates for a *S. aureus* vaccine is currently being investigated; in one study, live-attenuated small-colony variants have shown promising results compared to inactivated bacteria in murine models (84). However, in another study, a formalin-killed whole-cell vaccine candidate of *S. aureus* biofilm showed a significant reduction in the colonization of *S. aureus* in the udder in vaccinated mice compared to mice vaccinated with a vaccine candidate from planktonic *S. aureus* (85). A killed bacterin vaccine candidate against *S. aureus* was tested in primiparous gestating cows. There were no observations of any prevention of intramammary infection by *S. aureus* but a reduced multiplication of *S. aureus* in the mammary glands was observed (86). *S. aureus*' protein A has also been investigated as a possible vaccine target and a vaccine candidate has shown a significant reduction in bacterial load of the mammary glands of pregnant mice. However, the immunized mice were not protected when they subsequently were infected with biofilm producing encapsulated *S. aureus* (87). Currently, two mastitis vaccines are available on the market against *S. aureus* and *S. uberis* from the company HIPRA (Amer, Spain).

### *In vivo* Investigations of Biofilm in Bovine Mastitis

Most of the so-called *in vivo* investigations of biofilm in bovine mastitis have used experimental models (mice and sheep), and the majority of these studies focused on anti-biofilm treatment or vaccines against biofilm udder infections (Table 1) (44, 69, 70, 72, 83–85). Only a few studies investigated and confirmed biofilm *in vivo* within udder tissue of dairy cows with bovine mastitis. Two studies directly detected biofilm inside udders of dairy cows with mastitis. Clustering of *S. aureus* bacteria in udders of dairy cows with bovine mastitis were observed by microscopy to be located in the lumen of the alveoli and lactiferous ducts of the udders of experimentally infected dairy cows (47). In another study, the presence of biofilm was investigated directly in the udders of dairy cows by collecting swabs from the udders of slaughtered dairy cows with *S. aureus* infection. Swabs were obtained from the teat cistern, gland cistern, and parenchyma and were subsequently stained using immunofluorescence staining of polysaccharide intercellular adhesions (PIA), which is a component of the *S. aureus* biofilm matrix. The samples were investigated by fluorescence microscopy and PIA was found in 71 out of 184 swabs (46).

**Table 1:** Studies investigating biofilm forming bovine mastitis pathogens in animal models or the effect of possible antibacterial agents and vaccines in animal models.

**References**	**Year**	**Sample type**	**Focus of study**	**Pathogen**	**Experimental animal**
Cucarella et al. (88)	2001	Bovine subclinical mastitis and human isolates	Molecular basis of biofilm	*S. aureus*	Mice
Brouillette et al. (44)	2005	Clinical bovine mastitis isolates	Antibacterial treatment	*S. aureus*	Mice
Gogoi-Tiwari et al. (85)	2015	Bovine mastitis isolates	Vaccine	*S. aureus*	Mice
Collado et al. (83)	2016	Clinical bovine mastitis isolates	Vaccine	*S. uberis*	Mice
Gogoi-Tiwari et al. (87)	2016	Bovine mastitis isolates	Vaccine	*S. aureus*	Mice
Mordmuang et al. (70)	2019	Bovine mastitis isolates	Antibacterial treatment	*S. aureus*	Mice
Montironi et al. (89)	2019	Subclinical bovine mastitis isolates, milk samples	Investigation of phenotype, genotype and virulence	*Enterococcus faecium*	Mice
Côté-Gravel et al. (84)	2019	Bovine mastitis isolates	Vaccine	*S. aureus*	Mice
Marbach et al. (90)	2019	Subclinical bovine mastitis isolates, milk samples	Interactions between host and bacteria	*S. aureus*	Mice
Wang et al. (72)	2020	Isolates (Purchased strains)	Antibacterial treatment	*S. xylosus*	Mice
Prenafeta et al. (86)	2010	Ruminant mastitis isolates	Vaccine	*S. aureus*	Heifers, cows
Savijoki et al. (91)	2014	Bovine mastitis isolates	Genomics and proteomics	*S. epidermidis*	Cows
Seroussi et al. (92)	2018	Bovine mastitis isolates	Antibacterial treatment	*E. coli, S. aureus*	Cows
Cucarella et al. (40)	2004	Bovine subclinical mastitis isolates	Molecular basis of biofilm	*S. aureus*	Sheep

### The Bovine Mammary Immune Response to Biofilm Infection

The response to infections is crucial for the survival of mammals. The response mechanisms to bacterial and viral infections are widely investigated, however much less is known about the immune response toward biofilm. As per definition, host immune responses are tolerated by biofilms, and no specific anti-biofilm immune responses have been identified (82).

The protection against infectious agents in the bovine mammary gland has been recently reviewed by Sordillo (93). As for biofilm infections in general, the mammary gland response toward bacterial biofilms is not fully understood yet, and as described above very few *in vivo* investigations of mammary biofilm infections exist. Some studies have investigated the response of mammary cells to biofilm-producing strains of known mastitis-causing pathogens *in vitro*. The ability of *S. aureus* biofilm forming strains to adhere and invade the mammary cells is especially investigated. A study found that *S. aureus* biofilm showed lower invasion ability into mammary epithelial cells compared to planktonic *S. aureus* cultures and that the biofilm culture induced less cellular activation than the planktonic cultures. Both planktonic culture and *S. aureus* biofilm culture induced expression of interleukin 6 by mammary alveolar cells, which could be an anti-inflammatory response (94). This corresponds well to human research of biofilm infections and immune response, where biofilms do not trigger any specific immune responses (82) and downregulates specific virulence genes when the cell density is low to “fly under the radar” so the immune system does not detect the bacteria. Whenever the cell density is high enough, the bacteria can upregulate the virulence factors (95). However, other *in vitro* studies found no difference in the ability to invade host cells by non-biofilm producing mastitis strains compared to biofilm producing mastitis strains (96, 97). The role of the Bap protein expressed by *S. aureus* has been investigated in a lactating mouse model, where the surface protein Bap, involved in biofilm matrix, adhered to epithelial cells and bound to host receptor Gp96. The bacteria expressing the surface protein Bap did not invade the cells and had increased persistence in the mammary glands of the lactating mice, indicating that the protein promotes adhesion to the cells and limits invasion of the host cells (98).

The main question still not resolved is how the biofilms go undetected and survive the immune response (99) and more research is needed to answer that question both for mastitis and for all other biofilm infections. The current research is based on *in vitro* experiments and as discussed earlier in this review, more *in vivo* research is needed to fully understand the role of biofilm in mastitis.

## Discussion

The role of biofilm in the pathogenesis of bovine mastitis infection is still unclear. To the authors' best knowledge, only two papers investigate and detect the presence of biofilms inside udders from dairy cows with mastitis (46, 47). Plenty of *in vitro* studies investigate the biofilm forming abilities of mastitis pathogens isolated from milk samples. Similar to swabs and wound fluid samples, analyzing milk samples is a great, easy and quick way to investigate and culture the bacteria present in the samples and to determine, e.g., the genetic composition and antibiotic susceptibility. However, disadvantages are the risk of contamination from the environment and that it is only possible to detect bacteria present in or released into the milk, however bacteria embedded in the tissue, encapsulated bacteria, low-shedding bacteria, and potential biofilms might not be detectable in milk samples (100). Even if bacteria are isolated from milk samples and are able to form biofilm *in vitro*, this does not provide any information on the bacteria's phenotype *in vivo* in the infected udder. *In vitro* biofilms of *P. aeruginosa* have a markedly different genetic expression profile than *in vivo* biofilms during human infections (101). This is due to, e.g., the environment in the host tissue, interactions with the immune system, and antibiotic treatment that are impossible to fully mimic *in vitro*. Further, major physical differences exist between *in vitro* and *in vivo* biofilms; for example, *in vitro* biofilms normally form large mushroom-shaped structures, which are never observed *in vivo*, where the biofilms are markedly smaller in size (16).

Hence to find the actual role of biofilm in bovine mastitis, the approach needs to change from *in vitro* to *in vivo* investigations of biofilms in infected udders. When diagnosing biofilm infections in human medicine, the gold standard is to directly visualize the biofilms and concurrent immune response in the tissue. This can be done with, e.g., CLSM or scanning electron microscopy (15, 18, 102). Sample collection for biofilm diagnosis naturally varies for different diseases/tissue, e.g., from cystic fibrosis patients, expectorated sputum samples, bronchoalveolar lavage, or biopsies from removed lung tissues during lung transplantation can be collected (18, 102), and from chronic wounds, biopsies or debrided tissue can be investigated (103). Especially in wound infections, the spatial distribution of different bacterial biofilms within the tissue can be observed using microscopic examination; this method has further found the pathogen *P. aeruginosa* to be underestimated when performing culture of standard wound swabs (104, 105). A good technique to detect bacterial biofilms in tissue is peptide nucleic acid fluorescence *in situ* hybridization (PNA-FISH), with probes that hybridize to bacterial ribosomal RNA, which can subsequently be detected using CLSM. This is a sensitive method that is well-established in the research of biofilm infections in humans (102, 104, 106–108). This method would be applicable to udder tissue samples as well.

If biofilms are found present in mastitis udders, e.g., by use of the methods just described, the next question is whether the biofilms are part of the pathogenesis of bovine mastitis? Therefore, the immune response to the biofilms is also important to investigate. The cytological cure of mastitis is delayed compared to the bacteriological cure, meaning that when the infection appears cleared, the inflammation can continue in the udder (9). The cytological cure rate can be as low as around 20% and therefore it should be considered that chronic mastitis cases could be due to long-lasting inflammation, potentially driven by biofilms, after the apparent bacterial cure (9). The treatment of especially *S. aureus* mastitis cases is difficult, and therefore the connection between these cases and *S. aureus* biofilm presence and many virulence factors that are upregulated during mastitis infections should be further investigated (10, 11). We propose that udder cell and tissue models could potentially be applied to investigate how biofilms affect bovine udder tissue, however, studies of natural or experimentally induced mastitis will provide more information as a competent immune system would respond to the infection.

Collecting udder tissue biopsies from live dairy cows with mastitis for microscopy is difficult, if not impossible. However, biopsies can easily be obtained after euthanasia and by applying relevant staining and microscopy techniques, a more accurate view of biofilms' potential location and distribution as well as the related host immune response during mastitis can be revealed. Only a few papers provide information on biofilms' presence in udders from dairy cows with mastitis (46, 47) and more research is needed to elucidate biofilms' role in mastitis pathogenesis. Therefore, the collection of biopsies from euthanized animals might not have any direct clinical relevance, as the animals would be dead, but has important scientific relevance to better understand the disease and relate this to findings in milk samples. If biofilms play a role in bovine mastitis, diagnostic methods to detect biofilm in milk samples could be a possible way to easily diagnose the biofilms. However, for now, no such biofilm marker, specific biofilm product, or specific biofilm immune response have been identified that would be usable for quick and simple biofilm diagnostics neither in human or veterinary medicine. This is naturally the topic and aim of many human research groups' intense work, as biofilm infections play an important part of many human infections, and whenever found this would hopefully also be applicable to milk samples from bovine mastitis. By understanding the bacteria and biofilms including their interactions with the host immune system during mastitis infections, potentially new possible diagnostic methods could be developed as well as new optimized treatment options.

## Conclusions

Bovine mastitis is one of the most important diseases in the dairy industry and a better understanding of the role of biofilm in the disease is of high importance to achieve more successful treatments. Chronic biofilm infections are recognized as serious and difficult-to-treat diseases in human medicine. The majority of the research on biofilm and bovine mastitis has so far focused on *in vitro* studies; however, to uncover the presence of biofilm in udders of dairy cows suffering from mastitis, direct methods need to be applied. Some of the methods used in the diagnosis and research of biofilms in human infections could be applied to the research of biofilm in bovine mastitis. There is a need for *in vivo* research where the location and distribution of biofilms are investigated directly in the udder of dairy cows with mastitis and where these findings are related to findings in milk samples. The continuous unsuccessful antibiotic treatment of potential biofilm mastitis infections can increase the risk of antibiotic resistance, which is one of the biggest threats to human and animal health. The role of biofilm infections in bovine mastitis therefore seems a key to unlock the required knowledge to develop new diagnostic methods and treat the persistent and chronic cases of bovine mastitis.

## Methods

This review has included studies that examine biofilm in relation to mastitis in dairy cows. We have included studies investigating biofilm abilities, molecular properties, treatment options, prevention and interactions of bovine mastitis related pathogens. Studies published since 2000 were included. Reviews and manuscripts in other languages than English have not been included in this review.

The literature search was carried out using the database Pubmed on October 1st 2020 with the search words “bovine mastitis + biofilm.” Over 170 papers investigated the role of biofilm in bovine mastitis by *in vitro* methods and 16 papers used *in vivo* methods.

## Author Contributions

RP: original draft preparation, methodology, and writing. EJ: writing, editing, and supervision. VK, TB, KD-P, and RB: editing and supervision. All authors have read the manuscript and agreed to the published version.

## Conflict of Interest

The authors declare that the research was conducted in the absence of any commercial or financial relationships that could be construed as a potential conflict of interest.

## Nomenclature

CNS, Coagulase-negative staphylococci

CRA, Congo Red Agar

CLSM, confocal laser scanning microscopy

LAB, lactic acid bacteria

PIA, polysaccharide interstellar adhesions

PNA-FISH, peptide nucleic acid fluorescence *in situ* hybridization.
